# The benefits of treating undetectable tumors

**DOI:** 10.7554/eLife.09713

**Published:** 2015-08-05

**Authors:** Natalia L Komarova

**Affiliations:** Department of Mathematics, University of California, Irvine, Irvine, United Stateskomarova@uci.edu

**Keywords:** cancer prevention, evolution, mathematical models, human

## Abstract

Cancer prevention is predicted to result in more positive therapeutic outcomes than post-diagnostic interventions, and so may be a viable option for future personalized medicine.

**Related research article** Akhmetzhanov AR, Hochberg ME. 2015. Dynamics of preventive vs post-diagnostic cancer control using low-impact measures. *eLife* **4**:e06266. doi: 10.7554/eLife.06266

**Image** A mathematical model shows that the relapse time for tumors treated preventatively (blue) is larger than that for tumors treated by post-diagnostic interventions (red lines and yellow shading)

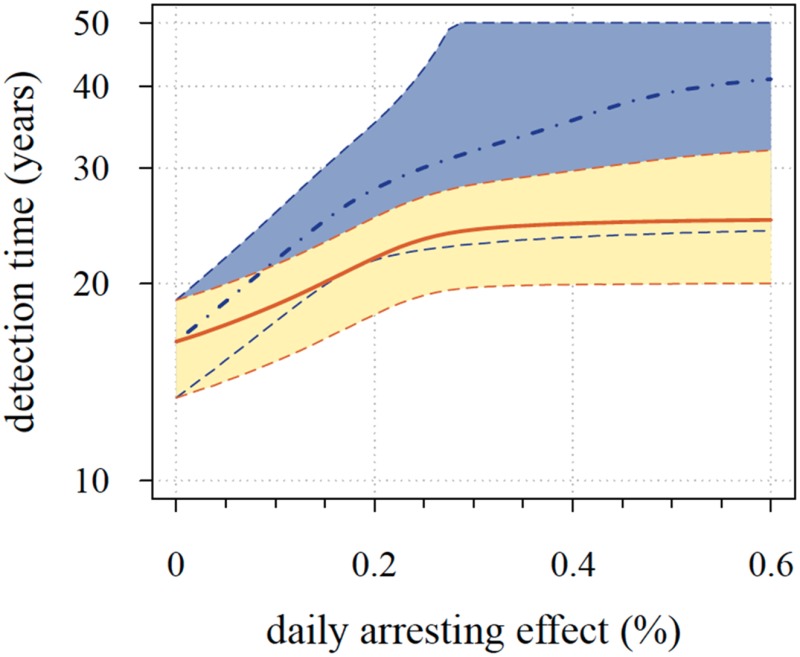

Many factors must be considered when choosing the most appropriate treatment strategy for a cancer patient, including drug availability, side effects, and the cost. Some scientists suggest that making this choice after the tumor has been detected may already be too late. Now, in eLife, Andrei Akhmetzhanov and Michael Hochberg at the University of Montpellier II have used a mathematical model to compare cancer prevention (treatment before a diagnosis) with cancer intervention (treatment after a diagnosis). In many cases, they found that prevention is the best option (Akhmetzhanov and Hochberg, 2015).

Cancer growth can be viewed as cell development and evolution that went astray. Certain mutations, dubbed ‘driver mutations’, alter the behavior of the cells, allowing them to divide faster or die slower; this results in faster overall growth (Haber and Settleman, 2007). This ability to grow more quickly, which depends on the genetic make-up of the cells as well as their environment, defines the cells' ‘fitness’. Thus, cells that are fitter than others can overcome the tightly regulated processes that keep the number of cells in an organ constant. These cells may then start to form a (pre)-malignant colony that can become highly diverse. As well as the driver mutations that increase cell growth, colony cells can also develop ‘passenger mutations’, which may be regarded as random modifications that may not cause a noticeable difference in the cells' behavior.

But that's until treatment begins. When a drug is applied, the environment inside the tumor changes, and all the cells that are sensitive to the treatment either slow down their growth or start decaying. However, it is possible that some of the passenger mutations that have been accumulating in the colony confer resistance to the drug. Cells with these mutations now experience a growth advantage because they are ‘fitter’ than the rest of the cells. This drug resistance is a serious problem in the treatment of cancer and has been studied extensively (Holohan et al., 2013). In particular, researchers have proposed mathematical models that aim to find a treatment strategy that minimizes the risk of drug resistance emerging (Goldie and Coldman, 2009; Komarova and Wodarz, 2014).

Akhmetzhanov and Hochberg have approached this old problem from a new angle. Using a model based on evolutionary dynamics, they compared the typical intervention strategies that are carried out post-diagnosis with a less common but promising strategy of tumor prevention. The logic behind this model is as follows. Imagine that a cancerous colony grows from one cell and randomly accumulates various mutations. In the post-diagnostic intervention strategy, the colony must have grown to a considerable size, such that it can be detected (a minimum of a billion cells). At this point, surgery is used to cut the colony down to a much smaller size, and then a certain anti-cancer treatment – for example, a chemotherapy drug – is applied. This treatment kills off the cells that are susceptible to it, but any resistant cells that have been generated in the colony will remain.

Alternatively, one can start treatment preventatively, even before the patient knows that cancer is growing. This is especially feasible for people that have a high likelihood of developing cancer at some point of their life; for example, because they have a inherited mutation that increases their cancer risk. In this second scenario, it is assumed that treatment hits the growing tumor before it has grown to a detectable size. For the sake of comparison, Akhmetzhanov and Hochberg assumed that preventative treatment starts when the colony is the same size as the colony that remains after surgery during post-diagnostic treatment. They also assumed that preventative treatment works in the same way as post-diagnostic treatments by removing susceptible, non-resistant cells. If the preventative treatment fails – that is, the colony still grows to a detectable size – surgery is carried out, followed by a second wave of treatment. The question is, which of the two treatment approaches leads to a higher probability of success? (In this model, success is defined as the tumor colony staying below a detectable size for at least 50 years.)

The results from Akhmetzhanov and Hochberg's model suggest that the preventative treatment strategy is often significantly better than the post-diagnostic treatment strategy, both in extending the tumor-free life of patients and in reducing the chance of treatment failure. Unexpectedly, the model also showed that preventative strategies can be highly effective, even at very low treatment doses.

The elegant mathematical methods used by Akhmetzhanov and Hochberg could be extended by future studies to include further subtleties of the cancer growth and treatment processes. For example, it will be important to examine the effects of cell competition and limited resources, as opposed to the unbounded growth considered so far. Furthermore, including different types of treatments – as well as combination treatments – will be important to obtain a more comprehensive picture of the relative benefits of the different treatment approaches. This is needed to account for the fact that long-term cancer prevention strategies may not use the same intensive chemotherapies that are currently used after cancer has been diagnosed. Finally, the toxicity effects experienced by patients on prevention therapies have to be studied and quantified in more detail.

These and other refinements will have to be implemented before the model can inform medical care professionals of the best drug doses and therapy start times to use on a patient. But the ground has been broken. The framework offered by Akhmetzhanov and Hochberg provides an important stepping-stone on our way to developing true personalized medicine.
